# Expanding the Repertoire of Gene Tools for Precise Manipulation of the *Clostridium difficile* Genome: Allelic Exchange Using *pyrE* Alleles

**DOI:** 10.1371/journal.pone.0056051

**Published:** 2013-02-06

**Authors:** Yen Kuan Ng, Muhammad Ehsaan, Sheryl Philip, Mark M. Collery, Clare Janoir, Anne Collignon, Stephen T. Cartman, Nigel P. Minton

**Affiliations:** 1 Clostridia Research Group, NIHR Biomedical Research Unit in GI Disease, Centre for Biomolecular Sciences, School of Life Sciences, University of Nottingham, Nottingham, United Kingdom; 2 Université Paris-Sud, Faculté de Pharmacie, Département de Microbiologie, Unité EA 40-43, Châtenay-Malabry, France; Institute Pasteur, France

## Abstract

Sophisticated genetic tools to modify essential biological processes at the molecular level are pivotal in elucidating the molecular pathogenesis of *Clostridium difficile*, a major cause of healthcare associated disease. Here we have developed an efficient procedure for making precise alterations to the *C. difficile* genome by *pyrE*-based allelic exchange. The robustness and reliability of the method was demonstrated through the creation of in-frame deletions in three genes (*spo0A*, *cwp84*, and *mtlD*) in the non-epidemic strain 630Δ*erm* and two genes (*spo0A* and *cwp84*) in the epidemic PCR Ribotype 027 strain, R20291. The system is reliant on the initial creation of a *pyrE* deletion mutant, using Allele Coupled Exchange (ACE), that is auxotrophic for uracil and resistant to fluoroorotic acid (FOA). This enables the subsequent modification of target genes by allelic exchange using a heterologous *pyrE* allele from *Clostridium sporogenes* as a counter-/negative-selection marker in the presence of FOA. Following modification of the target gene, the strain created is rapidly returned to uracil prototrophy using ACE, allowing mutant phenotypes to be characterised in a PyrE proficient background. Crucially, wild-type copies of the inactivated gene may be introduced into the genome using ACE concomitant with correction of the *pyrE* allele. This allows complementation studies to be undertaken at an appropriate gene dosage, as opposed to the use of multicopy autonomous plasmids. The rapidity of the ‘correction’ method (5–7 days) makes *pyrE* ^−^ strains attractive hosts for mutagenesis studies.

## Introduction

*Clostridium difficile* is the major cause of nosocomial diarrhoea and a major burden to healthcare services worldwide. The organism is resistant to various broad-spectrum antibiotics and capitalises on the disruption of the normal intestinal flora to cause disease symptoms ranging from asymptomatic carriage to a fulminant, relapsing and potentially fatal colitis [1], [2]. Sophisticated genetic tools to modify essential biological processes at the molecular level are pivotal in enabling the systematic study of the basis of colonization, virulence and pathogenesis of *C. difficile*. The requisite systems ideally need to be able to both make precise, *in situ* alterations to existing alleles, as well as introduce entirely new alleles to progress hypothesis driven research. The former include the generation of in-frame deletions and the introduction or correction of single or multiple nucleotide substitutions, deletions and insertions. The latter is required both to add entirely new, or altered alleles or provide the facility for the complementation of distal mutant alleles at chromosomal gene dosage.

For many years the required level of sophistication has been unavailable. Thus, directed mutants could only be made using insertional mutagens, reliant either on replication deficient [3] or defective [4], [5] plasmids, or on the deployment of the ClosTron and group II intron re-targeting [6], [7]. Recently, however, the cytosine deaminase gene (*codA*) of *Escherichia coli* was developed as a negative/counter selection marker for *C. difficile*, which enabled precise manipulation of the *C. difficile* chromosome for the first time [8]. In parallel, a second method (Allele-Coupled Exchange, ACE) has been formulated that allows the rapid insertion of heterologous DNA, of any size or complexity, into the genome [9]. Whilst a number of different genetic loci may be used to insert heterologous DNA via ACE, one exemplification of the method exploits the native *pyrE* gene, bringing about its inactivation by replacement of the wild type allele with a mutant allele lacking the codons from both the 5′ and 3′ end of the structural gene. The *pyrE* gene encodes orotate phosphoribosyltransferase [E.C.2.4.2.10]), which is an enzyme involved in *de novo* pyrimidine biosynthesis. It may be used as a positive/negative selection marker as it is essential in the absence of exogenous pyrimidines and it also renders 5-fluoro-orotate (FOA) toxic to cells. Toxicity occurs via a series of steps which result in misincorporation of fluorinated nucleotides into DNA and RNA and hence, cause cell death.

Mutant strains defective in *pyrE* created using ACE become auxotrophs requiring exogenous uracil to grow. They also lends themselves to the use of a functional *pyrE* allele as a negative/counter selection marker in a similar way to *codA* [8].The functionally equivalent gene, *URA5*, and *URA3* (encoding orotidine-phosphate decarboxylase) have been widely used as negative selection markers in *Saccharomyces cerevisiae* [10]. Indeed, this approach has allowed homologous gene replacement in a range of different microbes [11]–[14], including most recently *Clostridium thermocellum* [15] using the organisms own *pyrF* gene (equivalent to *URA3*) in a specifically created Δ*pyrF* mutant. Not unexpectedly, the Δ*pyrF* strain created required the addition of exogenous uracil to achieve equivalent cell density to the wild type in the rich media employed. With supplementation, whilst growth was slightly delayed, the eventual the growth rate was comparable to wildtype [15].

In this study, we have developed a procedure for the generation of allelic exchange mutants in *pyrE* mutants of two different strains of *C. difficile* (strain 630Δ*erm* and a BI/NAP1/PCR-Ribotype 027 strain, R20291) using a heterologous *pyrE* allele from *Clostridium sporogenes* as a negative/counter-selection marker. Use of a heterologous *pyrE* allele avoids homologous recombination with the native *pyrE* locus of *C. difficile*. The system has been used to make in-frame deletions in three different genes, *spo0A* (the master regulator of sporulation), *cwp84* (which encodes a cysteine protease) and *mtlD* (which encodes mannitol-1-phosphate 5-dehydrogenase). Crucially, having created these mutants, a specific ACE-vector is used to rapidly (within 5–7 days) restore the chromosomal *pyrE* allele to wild-type, allowing the specific in-frame deletion mutant to be characterised in a clean, wild-type background. Moreover, variants of the same vector may be used to deliver the wild-type allele of the deleted gene, either under the control of its native promoter or the strong *fdx* promoter, into the genome. The former allows complementation studies to be performed at an appropriate gene dosage, while the latter potentially allows the assessment of the effect of overexpressing the gene.

## Results

### Construction of a *pyrE^−^* mutant of R20291

**Figure 1:**
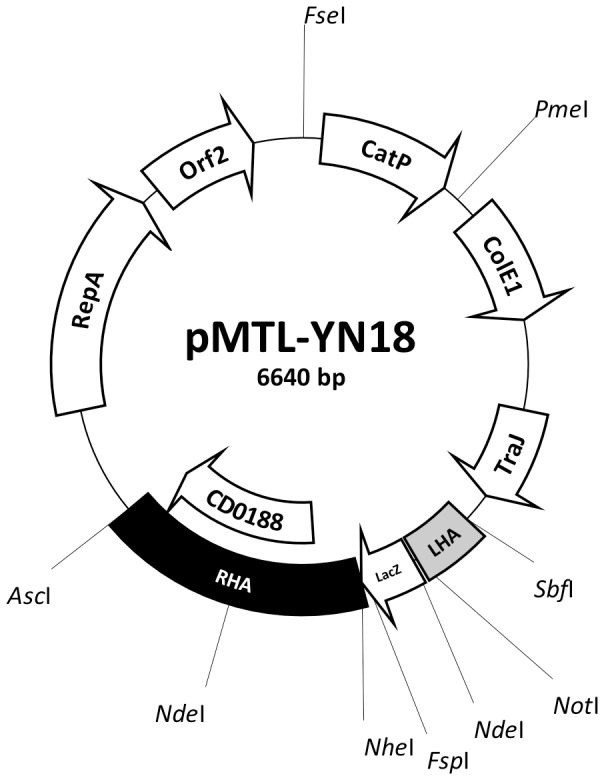
Plasmid pMTL-YN18. The ACE vector pMTL-YN18 is designed to create a deletion mutant specifically in the *C. difficile* strain R20191. Plasmid components are: CatP, the *catP* gene of *Clostridium perfringens* conferring thiamphenicol resistance; ColE1, the replication region of the *E.coli* plasmid ColE1; TraJ, transfer function of the RP4 *oriT* region; RepA and Orf2, the replication region of the *Clostridium botulinum* plasmid pBP1; LHA, left-hand homology arm encompassing a 300 bp internal fragment of the R20291 *pyrE* gene lacking 50 nucleotides from the 5′-end, and 235 bp from the 3′-end; RHA, right-hand homology arm comprising encompassing the 1200 bp region of DNA immediately downstream of *pyrE*, and; *lacZ'*, gene encoding the alpha fragment of the *E.coli* β-galactosidase (and containing a multiple cloning site region derived from plasmid pMTL20 [19]).

The *pyrE^−^* mutant of strain 630Δ*erm* was previously made using Allele-Coupled Exchange (ACE) and the purpose built, replication defective vector pMTL-JH18::lambda6.5 [9]. It is based on the replicon of plasmid pCB102 [16], previously shown to be the least effective (most unstable in terms of segregation into daughter cells) in strain 630Δ*erm* of the four replicons available in the pMTL80000 modular vector series [17]. In strain R20291, however, the plasmid pBP1 replicon is the most defective, *ie.*, plasmids that use this replication region are more rapidly lost in the absence of antibiotic selection [18]. Accordingly, pMTL-YN18 was constructed in order to generate a *pyrE* mutant of *C. difficile* R20291 (Figure 1). pMTL-YN18 is equivalent to the pMTL-JH18::lambda6.5 vector used to construct a Δ*pyrE* mutant in *C. difficile* 630Δ*erm*, except the pCB102 replicon is substituted with that of pBP1 and the 6.5 kb fragment of phage lambda DNA is absent. It carries two asymmetric homology arms that flank a *lacZ'* multiple cloning site (MCS) region. The shorter, 300 bp left-hand homology arm (LHA) is composed of a variant *pyrE* allele that lacks 50 nucleotides from the 5′-end of the gene and is devoid of 235 bp from the 3′-end of the gene. The larger 1200 bp right-hand homology arm (RHA) encompasses the region residing immediately downstream of the *pyrE* gene in the *C. difficile* genome.

Plasmid pMTL-YN18 was used to generate a *pyrE* mutant of *C. difficile* R20291. Five independent fluoroorotic acid (FOA) resistant colonies were chosen and shown to be *pyrE* deletion mutants. Thus, they all required exogenous uracil for growth on minimal media, were no longer thiamphenicol resistant, consistent with plasmid loss, and generated a PCR fragment of the expected size (623 bp) using primers that flanked the *pyrE* locus (Supporting Information File S1). Nucleotide sequence of the 623 bp fragment generated confirmed the presence of the expected deletion.

### Restoration of *pyrE*+ phenotype in 630Δ*erm* and R20291

**Figure 2:**
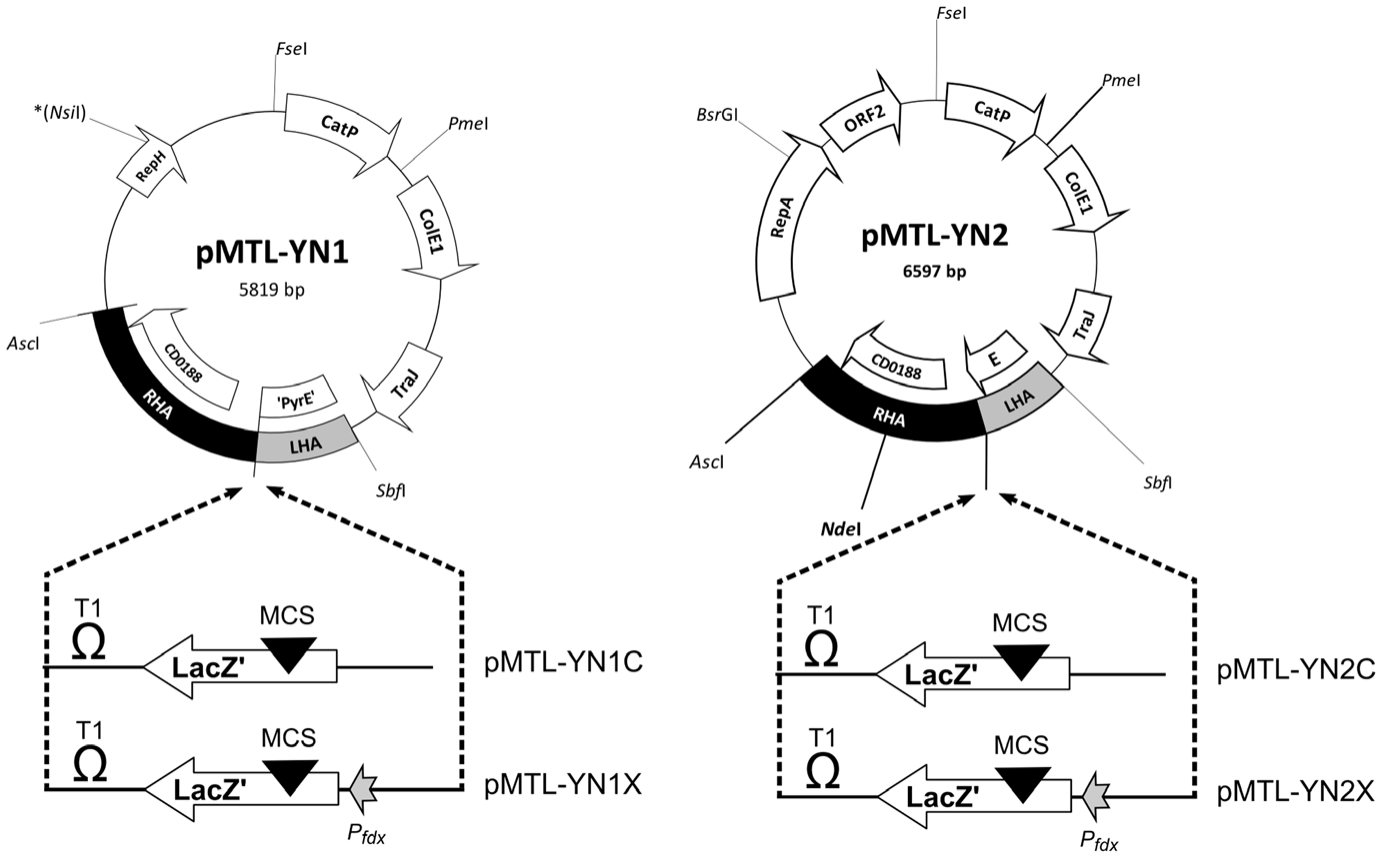
PyrE ACE correction vectors for *C. difficile* 630Δ*erm* (pMTL-YN1) and R20291 (pMTL-YN2). Both vectors carry identical components between their FseI and SbfI restriction sites. These are: CatP, the *catP* gene of *Clostridium perfringens* conferring thiamphenicol resistance; ColE1, the replication region of the *E.coli* plasmid ColE1, and; TraJ, transfer function of the RP4 *oriT* region. Plasmids pMTL-YN1C and pMTL-YN2C have an additional segment of DNA inserted between the left-hand homology arm (LHA) and the right-hand homology arm (RHA) which carries: a transcriptional terminator (T1) of the ferredoxin gene of *Clostridium pasteurianum*; a copy of the *lacZ'* gene encoding the alpha fragment of the *E.coli* β-galactosidase, and; a multiple cloning site (MCS) region derived from plasmid pMTL20 [19]. Plasmids pMTL-YN1X and pMTL-YN2X differ from pMTL-YN1C and pMTL-YN2C, respectively, in that they carrying the promoter region (P*_fdx_*) of the *Clostridium sporogenes* ferredoxin gene.

With *pyrE* mutants of both *C. difficile* 630Δ*erm* and R20291, it was desirable to demonstrate that they could be converted back to uracil prototrophy, through restoration of the *pyrE* locus to wild-type. Accordingly, ACE plasmids pMTL-YN1 and pMTL-YN2 were constructed to correct the *pyrE* mutations in *C. difficile* 630Δ*erm* and R20291, respectively (Figure 2). These plasmids were broadly equivalent to pMTL-JH14, used to correct a similar *pyrE* mutation in *C. acetobutylicum* [9], with a *pyrE* allele that lacked the first 50 nucleotides of the open-reading-frame forming the short 300 bp LHA. Crucially, they differed from pMTL-JH14 in that they did not carry the *lacZ'* region present in this plasmid between the LHA and RHA. As a consequence, the strains generated using them would be indistinguishable from the wild-type at the *pyrE* locus. Both plasmids were conjugated into their respective *pyrE* mutants from an *E. coli* CA434 donor and, following the procedure outlined in MATERIALS AND METHODS, several dozen colonies were obtained that were able to grow without uracil supplementation. Six random colonies from each strain were screened by PCR using primers which annealed to the chromosomal genes that reside up- and down-stream of the *pyrE* gene. In all cases, the expected 2,058 bp band was generated, equivalent to the wild-type control, whereas no band was generated when the template DNA was derived from the *pyrE* mutant (Supporting Information File S1). Nucleotide sequencing of the fragment generated confirmed that the cells carried a wild type *pyrE* gene.

### Formulation of the components of an Allelic Exchange vector

Aside from the gene specific knock-out cassettes, the two most important components of the desired allelic exchange vector are the *pyrE* allele, to be used as the negative/counter-selection marker, and the clostridial plasmid replicon. In the case of the *pyrE* allele, it is desirable that the gene used is heterologous, to avoid recombination at the native chromosomal *pyrE* locus of *C. difficile*. Therefore, we selected the *pyrE* gene of *Clostridium sporogenes*, which shares only 65% identity with its *C. difficile* equivalent. The *pyrE* gene was cloned into pMTL83151 and the resultant plasmid (pMTL-ME2, Figure 4) shown to be able to restore the *C. difficile pyrE* mutants to prototrophy, *ie.*, they were able to grow on minimal media lacking uracil supplementation (data not shown).

**Figure 3:**
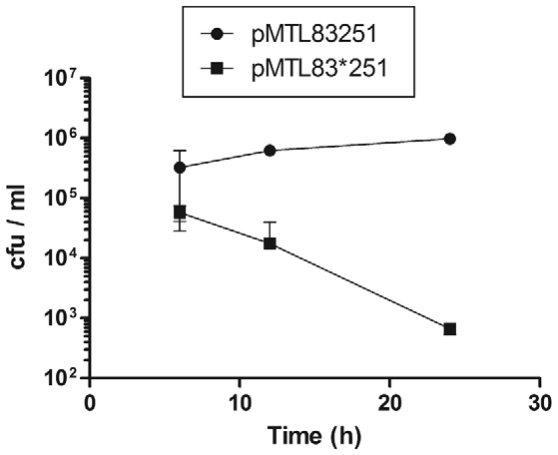
Segregational stability of pMTL83*251 or pMTL83251 in *C. difficile* strain 630Δ*erm*. The two plasmids differ only in that pMTL83*251 has a frame shift in the pCB102 RepH gene, introduced by blunt-end ligation following cleavage with NsiI. Cells carrying the two plasmids were grown in BHIS media in the absence of antibiotic and then CFUs estimated on agar media supplemented with thiamphenicol after 6, 12 and 24 h of growth. The illustrated data was derived from three independent experiments.

The isolation of the initial, single cross-over plasmid integrants required for the creation of allelic exchange mutants is facilitated by the use of ‘pseudo-suicide’ vectors [8], [18]. These are plasmids carrying *catP* (encoding resistance to thiamphenicol) that are sufficiently replication defective for there to be a significant growth disadvantage in the presence of thiamphenciol compared to cells in which the plasmid, together with *catP*, has integrated. Such integrated clones grow faster, and produce larger colonies, on agar media supplemented with thiamphenicol, because all of the progeny carry a copy of *catP*. Cells carrying *catP* on a non-integrated, defective autonomous plasmid, in contrast, grow slower because in the presence of antibiotic they are limited by the rate at which the plasmid is segregated amongst the daughter population. Previously, the pCB102-based plasmid had been made more defective in strain 630Δ*erm* by increasing the size of the final vector through the insertion of 6.5 kb of DNA derived from phage lambda [9]. Here we took a different strategy, and explored the effect of introducing a frame-shift mutation into the 3′-end of putative *repH* gene of the pCB102 replication region. Accordingly, the modular plasmid pMTL83251 was cleaved with NsiI, treated with T4 polymerase and subjected to self-ligation, yielding pMTL83*251. Nucleotide sequencing confirmed that the expected modification had occurred, altering the sequence ATGCAT to AT. The resulting deletion of 4 nucleotides (TGCA) causes a frame-shift in the coding sequence, replacing the COOH-terminal region of RepH (CIKYYARSFKKAHVKKSKKKK) with LNIMGALKKLM. The effect on segregational stability was tested by growing cells of 630Δ*erm* carrying either pMTL83251 or pMTL83*251 in the absence of antibiotic selection and measuring plasmid loss. The results are shown in Figure 3, and demonstrate that the frame-shift has caused a significant reduction in the segregational stability of the plasmid.

**Figure 4:**
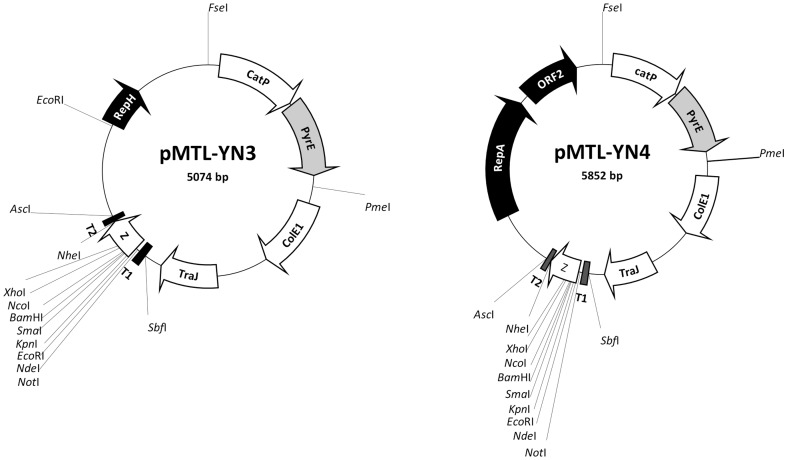
Allelic Exchange vectors for manipulation of *C. difficile* 630Δ*erm* (pMTL-YN3) and R20291 (pMTL-YN4). Common plasmid components are: CatP, the *catP* gene of *Clostridium perfringens* conferring thiamphenicol resistance; PyrE, the *pyrE* gene of *Clostridium sporogenes*; ColE1, the replication region of the *E.coli* plasmid ColE1, and; TraJ, transfer function of the RP4 *oriT* region; Z, the *lacZ'* gene encoding the alpha fragment of the *E.coli* β-galactosidase (and containing a multiple cloning site, MCS, region derived from plasmid pMTL20); T1, a transcriptional terminator isolated from downstream of the *Clostridium difficile* strain 630 CD0164 gene, and; T2, a transcriptional terminator of the ferredoxin gene of *Clostridium pasteurianum*. The position of the frame-shift generated at the NsiI site is indicated by an asterick. Plasmid pMTL-ME2 is identical to plasmid pMTL-YN3, except it carries an NsiI site at the 3′-end of RepH at the position marked by an asterick.

Accordingly, the NsiI site of plasmid pMTL-ME2 was similarly frame-shifted, and the resulting plasmid was designated pMTL-YN3 (Figure 4). For manipulations in R20291, the pCB102 replication region of pMTL-YN3 was simply replaced with that of pBP1, by cleaving pMTL-YN3 with AscI and FseI and replacing the excised fragment carrying the pCB102 replicon with the equivalent fragment from the pBP1-based plasmid pMTL82251 [17]. The resulting plasmid was designated pMTL-YN4 (Figure 4).

### Exemplification of the System to create *spo0A* mutants of strains 630Δ*erm* and R20291

To test the system, we made in-frame deletions in the *spo0A* gene of both *C. difficile* 630Δ*erm* and R20291 using plasmids pMTL-YN3::630spo0A* and pMTL-YN4::R20291spo0A*, respectively, as detailed in the allele exchange procedure outlined in MATERIALS AND METHODS. After two (630Δ*erm*) and four (R20291) passages of the transconjugants on BHIS selective media, one of the six visibly larger colonies derived from 630Δ*erm*, and four of the six visibly larger colonies derived from R20291, were found to be pure single crossover mutants by PCR (data not shown). Subsequently, single colonies were re-streaked onto minimal medium supplemented with FOA and uracil to select for cells in which the integrated plasmid had excised. The isolated FOA resistant colonies (four in the case of 630Δ*erm* and twelve in the case of R20291) were then screened by PCR using primers spo0A-YN-F2 and spo0A-YN-R2 that anneal to the upstream and the downstream sequence of *spo0A*, respectively. Of the four 630Δ*erm* colonies screened, one yielded the expected 1,845 bp DNA fragment, indicative of an in-frame deletion (Supporting Information File S1). The other colonies yielded a 2,331 bp DNA fragment, consistent with the presence of a wild-type copy of the gene. In the case of R20291, two of the twelve colonies screened yielded the 1,845 bp DNA fragment indicative of an in-frame deletion (Supporting Information File S1). Nucleotide sequencing of the 1,845 bp DNA fragments confirmed that the expected allelic exchange event had taken place in all three putative mutants.

The sporulation minus phenotype of the mutants was confirmed by assaying colony forming units (cfu) on BHIS supplemented with 0.1% [w/v] sodium taurocholate before and after heat shock (65°C for 30 min), as previously described [20]. In each case, no colonies were obtained after heat shock, whereas the wild type controls gave counts of 1.28×10^4^ and 9.17×10^4^ cfu for CRG2548 (R20191Δ*pyrE*) and CRG2547 (630Δ*erm*Δ*pyrE*), respectively. Phase contrast microscopy confirmed that unlike the wild type strains, phase-bright spores were absent in the mutant cultures.

### Creation and *in situ* complementation of in-frame deletion mutants of *cwp84*

To further test the system, we constructed mutants of the *cwp84* gene of both 630Δ*erm* and R20291. Cwp84 is a cysteine protease responsible for the post-translational cleavage of SlpA into the two proteins High Molecular Weight (HMW) and Low Molecular Weight (LMW) SlpA, which are the major components of the *C. difficile* surface layer. ClosTron mutants of *cwp84* have been made elsewhere in *C. difficile* 630Δ*erm* [21], [22], and in the one study [22] complemented using an autonomous plasmid. *cwp84* in-frame deletion mutants were constructed following an identical procedure to that used to isolate the *spo0A* mutants, using plasmids pMTL-YN3::630cwp84* and pMTL-YN4::R20291cwp84*. One of the three 630Δ*erm* FOA resistant colonies screened yielded a 1,218 bp PCR product, indicative of the desired in-frame deletion (Supporting Information File S1). In the case of R20291, three of four FOA resistant colonies screened yielded the 1,218 bp PCR product, indicative of the desired in-frame deletion (Supporting Information File S1). Nucleotide sequencing of the 1,218 bp fragments confirmed that the desired in-frame deletion had been obtained.

**Figure 5:**
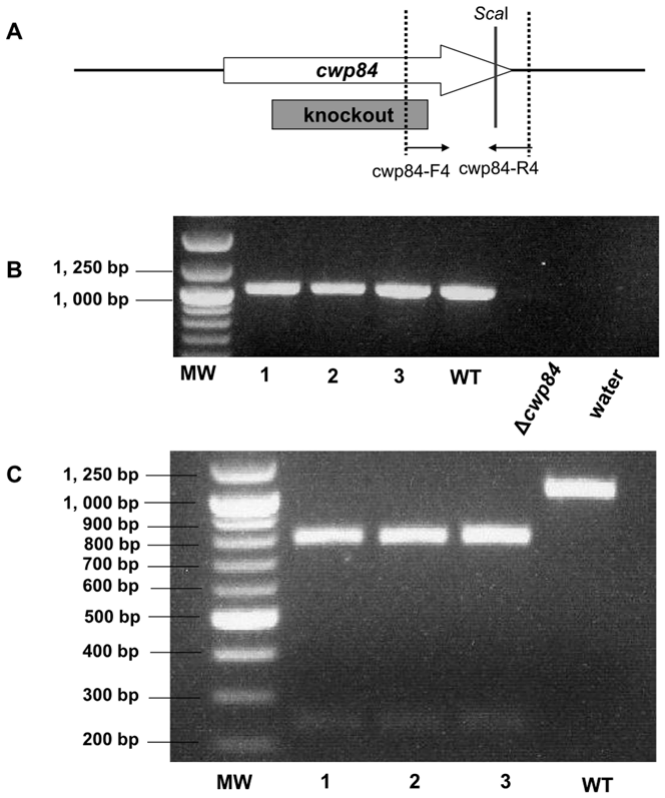
PCR screening of double crossover candidate clones for complementation of the *cwp84* gene in *C. difficile* 630 Δ*erm* Δ*cwp84*. (A) Schematic diagram of the complementation of *cwp84*, with a single nucleotide change to base 2280 of *cwp84* from a T to an A, without changing the corresponding valine amino acid residue and at the same time creating a *Sca*I site. The purpose of this single nucleotide change was to prove the occurrence of the complementation event. (B) PCR screening of candidate clones of the complemented *cwp84* gene. Primers cwp84-F4 and cwp84-R4 anneal to the internal sequence of the knockout cassette and the downstream sequence of *cwp84*, respectively, resulting in a 1, 026 bp PCR product from double-crossover complemented clones and wild-type, while no PCR product is expected from Δ*cwp84* mutants. MW is a 2-Log DNA Ladder (NEB) molecular weight marker, WT is a wild-type *C. difficile* DNA control, and 1–3 are the candidate clones. All candidates 1 to 3 show the expected complemented 1, 026 bp band, thereby confirmed as *cwp84* complemented clones, as seen in the wildtype control. (C) PCR products amplified using primers cwp84-F4 and cwp84-R4 from candidates clones and wildtype were analysed by RE digestion with *Sca*I. PCR products amplified from cwp84 complemented clones were cut into two fragments (786 and 240 bp), whereas PCR products amplified from the wildtype control did not.

A second round of *pyrE*-based allele exchange was carried out in the 630Δ*erm* and R20291 *cwp84* mutants, using pMTL-YN3::630*cwp84^c^* and pMTL-YN4::R20291*cwp84^c^*, respectively, to restore the mutant alleles back to wild-type, *in situ*. A conservative base substitution was made to introduce a ScaI restriction site into the restored *cwp84* sequence (Figure 5A). Three 630Δ*erm* FOA resistant colonies were screened and yielded a 1.1 kb PCR product with primers cwp84-F4 and cwp84-R4, indicating that the *cwp84* allele had been successfully restored (Figure 5B). Subsequent digestion of the PCR products with ScaI confirmed that restoration of the *cwp84* allele had been successful in each of the three clones screened (Figure 5C). Similar analysis was carried out on nine R20291 FOA resistant clones. Successful restoration of *cwp84* was confirmed in each of the nine clones, as evidenced by a 1.1 kb PCR product which was cleaved by Sca I (data not shown).

**Figure 6:**
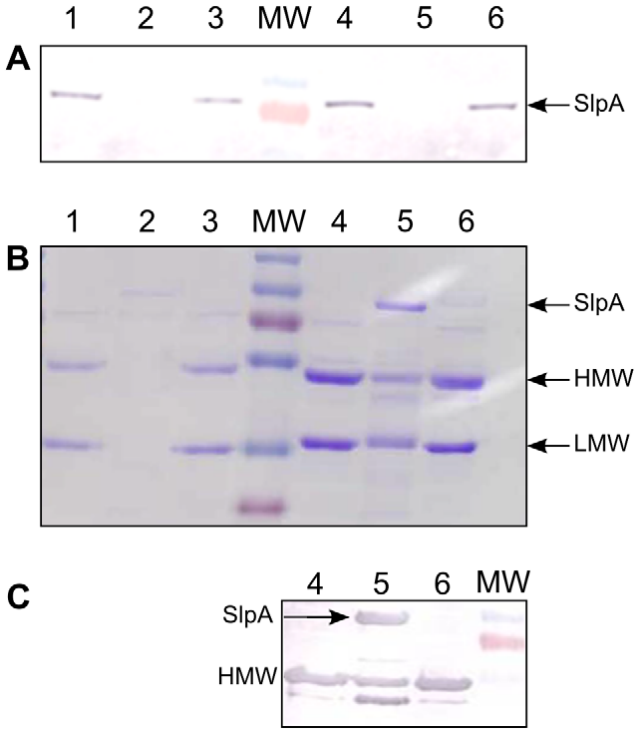
Glycin extracts analysis of 630Δerm and R20291 WT, mutant and complemented strains. (A) Immunoblot analysis with anti-Cwp84 antibodies of glycin extracts, showing complete absence of Cwp84 in the mutants compared to WT and complemented strains. (B) SDS-PAGE of glycin extracts of 630Δerm and R20291 WT, mutant and complemented strains, showing no processing of SlpA precursor in the 630Δ*cwp84* mutant, and in contrast, an incomplete processing of SlpA in the R20291Δ*cwp84* mutant. (C) Identification of the HMW-SLP in the glycin extract of the R20291Δ*cwp84*, showing that a partial processing of SlpA takes place in this mutant even in absence of the Cwp84 protease. Lanes 1, 630Δ*erm*; lanes 2, 630Δ*erm*Δ*cwp84*; lanes 3, 630Δ*erm*Δ*cwp84* complemented; lanes 4, R20291; lanes 5, R20291Δ*erm*Δ*cwp84*; lanes 6, R20291Δ*erm*Δ*cwp84* complemented; MW, molecular weight standard.

Before carrying out any phenotypic characterisation, the *cwp84* mutants and their restored derivatives were converted back to a *pyrE*-positive (uracil prototrophy) phenotype using pMTL-YN1 and pMTL-YN2 for the 630Δ*erm*-derived strains and the R20291-derived strains, respectively. In keeping with previous observations [21], [22], the mutants grew poorly on anaerobic blood agar compared to the wild-type and the complemented strains (Supporting Information File S1). S-layer extracts from the WT, mutant and complemented strains were prepared by the glycin acid method. The absence of the Cwp84 protease in the 630Δ*erm* and R20291 mutant strains was confirmed by immunoblot analysis with anti-Cwp84 antibodies (Figure 6A). Furthermore, while glycin extracts of both the 630Δ*erm* wild type strain and the complemented mutant contained two bands corresponding to the cleaved HMW and LMW S-layer proteins, only a band corresponding to the unprocessed SlpA precursor was evident in the *cwp84* mutant strain (Figure 6B). Interestingly, whilst broadly equivalent results were obtained with the R20291 strains, it was clear that the *cwp84* mutant contained two lower intensity bands, most likely corresponding to the HMW and LMW, in addition to the SplA precursor (Figure 6B). The identity of the HMW-SLP band was further confirmed by immunoblot (Figure 6C). This suggests that some proteolytic cleavage of SlpA may have occurred in the R20219 mutant, despite the absence of Cwp84.

### Creation and rapid *in trans* complementation of in-frame deletion mutants

Whilst the *in situ* complementation of mutant alleles represents the ‘gold standard’, the procedure required is lengthy (approx. 3–4 weeks), and therefore adds to the time needed to characterise a mutant. An alternative strategy would be, having made a specific in-frame deletion in a *pyrE* strain, to use ACE to introduce a wild type copy of the gene into the chromosome at the *pyrE* locus concomitant with the correction of this allele back to a PyrE-positive phenotype. Accordingly, complementation vectors (pMTL-YN1C and pMTLYN2C, for strains 630 and R20291, respectively) were constructed (MATERIALS AND METHODS) especially for this purpose, together with derivatives (pMTL-YN1X and pMTLYN2X, for strains 630 and R20291, respectively) carrying a strong promoter (the *fdx* promoter) to direct the over expression of the complementing gene. In contrast to *in situ* complementation, and as a consequence of being able to directly select for uracil prototrophy without the need to isolate pure single crossover clones, the successful introduction of genes using these vectors takes between 5 and 7 days.

To test the utility of this approach, we targeted the *mtlD* gene which forms part of the operon responsible for mannitol metabolism in *C. difficile* strain 630Δ*erm*, using plasmid pMTL-YN3::630mtlD. The ability of *C. difficile* to ferment mannitol is a distinguishing feature and forms the basis of CDSA (*Clostridium difficile* Selective Agar) developed by Becton Dickinson [23]. Fermentation of mannitol in this medium causes a pH drop, which causes the indicator present to change in color, from red to yellow. In many bacteria mannitol is transported into the cell via a typical phosphotransferase system (PTS), and therefore couples transport to phosphorylation of the sugar [24]. The *mtl* operon in *E. coli* consists of the *mtlA*, *mtlR*, and *mtlD* genes that respectively encode the mannitol transporter (enzyme IICBA*mtl*), a transcriptional regulator, and mannitol-1-phosphate dehydrogenase [25]. The arrangement of genes in *C. difficile* 630Δ*erm* is predicted to be equivalent to that of *B. stearothermophilus* and comprises four putative genes: *mtlA* (enzyme IICB*mtl*), *mtlR*, *mtlF* (enzyme IIA*mtl*), and *mtlD* [26], [27].

Following transfer of the knock-out plasmid pMTL-YN3::630*mtlD* into *C. difficile* 630Δ*erm*, three FOA resistant clones were isolated. PCR screening using primers mtlD-F3 and mtlD-R3, that anneal to the upstream and the downstream sequence of *mtlD*, demonstrated that two clones were Δ*mtlD* mutants, yielding a PCR product of 1,418 bp, while the third clone was a wild type revertant, yielding a PCR product of 2,582 bp (Supporting Information File S1). Nucleotide sequencing of the 1,418 bp products confirmed that the desired *mtlD* in-frame deletion had been created.

On isolation of the *mtlD* mutant, the *pyrE^−^* gene was converted back to *pyrE^+^* using plasmid pMTL-YN1 as described above. In addition, two further derivatives of pMTL-YN1 were employed which simultaneously delivered a functional copy of the *mtlD* wild type gene into the genome immediately downstream of the corrected *pyrE* gene. Plasmid pMTL-YN1C*mtlD* introduced the *mtlD* structural gene into the chromosome together with its native promoter. In contrast, pMTL-YN1X*mtlD* placed the inserted gene under the control of the strong *fdx* promoter. In both instances, the inserted *mtlD* gene was followed by the transcriptional terminator sequence [6] derived from the ferredoxin gene of *Clostridium pasteurianum*. The purpose of the terminator was to prevent polar effects being exerted by the inserted gene on the downstream gene, CD0188. In both cases, six independent PyrE+ clones able to grow on minimal media in the absence of uracil were screened for the presence of the wild type allele by PCR using primers Cdi630-pyrD-sF1 and Cdi630-CD0189-SR3. All twelve clones gave the expected 2,058 bp DNA product, equivalent to a wild type control. Nucleotide sequencing confirmed its identity as a wild type *pyrE* allele.

**Figure 7:**
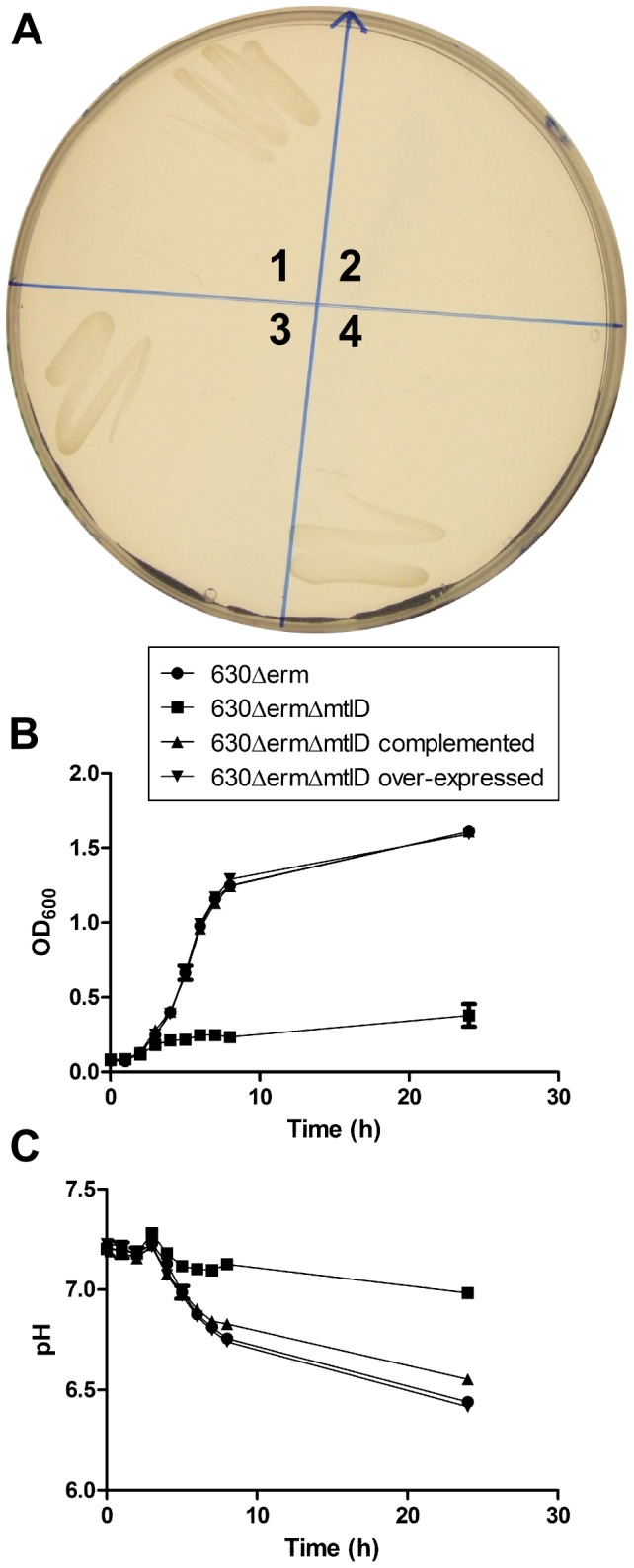
Growth of *C. difficile* 630Δerm strains with mannitol as the sole carbon source. (A) Clock-wise from top-left, *C. difficile* 630Δ*erm* (1) 630Δ*erm*Δ*mtlD* mutant (2), and 630Δ*erm*Δ*mtlD*-complemented (3) and 630Δ*erm*Δ*mtlD*-overexpressed (4) were streaked onto minimal media agar with mannitol as the sole carbon source and incubated for 48 h to observe growth. In contrast to the wild type, complemented and overexpressed strains, no growth was evident for the 630ΔermΔ *mtlD* mutant. (B) The growth of Δ*mtlD* was limited in mannitol broth, while growth of the Δ*mtlD* complemented and *mtlD* overexpressed strains were restored to wildtype levels. (C) The pH of the media broth showed a dip in pH caused by the fermentation of mannitol for the wildtype, Δ*mtlD* complemented and Δ*mtlD* overexpressed strains, which correlate to their growth. The 630 Δ*erm* Δ*mtlD* mutant strain grew very weakly in mannitol broth, which was reflected in the sustained pH levels of the media. All experiments were undertaken in triplicate. The data, complete with error bars is provided in the Supporting Information File S1.

Phenotypic analysis confirmed that the *mtlD* mutant was no longer able to ferment mannitol. Thus, in contrast to the wild type and complemented strain, no growth was evident on minimal media containing mannitol as the sole carbon source (Figure 7A). Moreover, growth of the mutant in liquid media comprising a mannitol-rich complex media (BD Diagnostics, USA) was severely retarded (Figure 7B), and only a slight decrease in the pH of the culture was evident (Figure 7C). The complemented mutant, on the other hand grew at the same rate as the wild type, as did the complemented mutant in which the native *mtlD* promoter had been replaced with the strong *fdx* promoter. In this instance, at least, any phenotypic benefit of increasing the expression of *mtlD* could not be measured.

Finally, to confirm the utility of this approach to complementation, we created the complementation plasmids pMTL-YN1C*spo0A* and pMTL-YN2C*spo0A* (MATERIALS AND METHODS) and used them to insert a functional copy of the strain 630Δ*erm spo0A* gene into the genomes of the *spo0A* mutants of strains 630Δ*erm* and R20291 concomitant with restoration of the *pyrE* mutant allele back to wild type. The sporulation phenotypes of the resultant strains were restored to that of the wildtype (Supporting Information File S1).

## Discussion

For many years the gene tools available to bring about specific, directed modifications of *C. difficile* at the genetic level were scant indeed. In the present study we have capitalised on the development of ACE technology to devise and exemplify methods for the generation and complementation of precise allelic exchange mutants in the *C. difficile* genome based on the use of various *pyrE* alleles. The use of genes involved in uracil metabolism, such as *pyrE* and *pyrF*, as a negative-/counter- selection marker has previously been demonstrated in a number of different bacteria and yeast [10]–[15], where it is reliant on the initial creation of a mutant defective in the chosen gene to act as the host for mutant construction. The deployment of ACE technology ensures this is a relatively simple and rapid undertaking. More important, as demonstrated here, ACE may be used to rapidly convert the host back to *pyrE^+^*, allowing the assessment of the mutant phenotype in a ‘clean’, wild type background. Such an undertaken has not generally been applied in other systems. Thus, for instance, the mutants created in *C. thermocellum* [15] using the organisms own *pyrF* gene as a counter-selection marker were always analysed in the Δ*pyrF* strain in which they were created. Similarly, a mutagenesis system developed in *Bacillus subtilis*, based on the use of the *upp* gene [28], has been adapted for use in *C. acetobutylicum* (P Soucaille, pers comm.) and is always undertaken, and the resultant mutants made analysed in, a *upp-* background. The use of a background defective in nucleoside metabolism can complicate the assessment of the effect of the mutation under analysis. This is particularly the case in the investigation of pathogens and virulence, where mutants in nucleoside metabolism are considered a form of disablement in their own right [29]. This has considerable impact on studies designed to assess the effect of mutation of other loci on virulence, particularly *in vivo*.

We have demonstrated the robustness and reliability of this *pyrE* method of mutagenesis in both *C. difficile* 630 Δ*erm* and *C. difficile* R20291, through the creation of in-frame deletion mutants in *spo0A*, *cwp84* and *mtlD*. We have re-affirmed the value of strategies based on pseudo-suicide vectors, and in the case of 630Δ*erm* improved the options available through the further disablement of the pCB102 replicon used by the introduction of a frameshift in the *repH* gene. This was found to facilitate the ease with which single crossover integrants were isolated, which tend to emerge after just two passages of transconjugants on selective media. This is in contrast to strain R20291 allelic exchange vectors, which make use of the pBP1 vector. During the course of this work we have confirmed that the annotated *mtlD* locus is indeed involved in mannitol utilisation and re-affirmed that Cwp84 plays a role in the processing of SlpA. Intriguingly, we found that despite the introduction of the *cwp84* frame-shift in R20291, unlike 630Δ*erm*, some processing of SlpA was still occurring in this strain. The in-frame deletion of both strains results in an internal deletion of 406 amino acids from the protein (total 804 amino acids). Whilst the truncated protein that results still contains Cys116, an important residue in the active site of the protease [22], it seems unlikely that the protein retains proteolytic activity, particularly as the truncated protein in the 630Δ*erm* mutant is identical. Moreover, no signal was detected by immunoblot analysis in the two mutant strains, suggesting that there is no expression at all of the protease, even in a truncated form, or that this form is quickly degraded in the cell. Perhaps strain R20291 produces a secondary protease also able to process SlpA? At this stage no one explanation can be ruled out. Here our focus has been on the development and description of the mutagenesis method, and our data on individual mutants remains observational.

The method developed here is in many respects equivalent to our recent description of the use of *codA* as a counter selection [8]. Indeed, the allelic exchange cassettes and methodological procedures are the same, except counter-selection is based on differential resistance to FOA (PyrE) rather than FC (CodA). Accordingly, the isolation of mutants using either protocol takes around 3–4 weeks to accomplish once the allelic exchange vectors have been constructed, and both methods may be subsequently employed to undertake *in situ* complementation of the mutated allele (exchange of the mutant allele with the wild type gene). A pivotal difference is that *codA*-based mutagenesis [8] can be undertaken in a wild type background (provided the host does not carry a native *codA* gene, as is the case in *C. botulinum*, *C. sporogenes* and *C. ljungdahlii*), whereas the method described here requires the initial creation of a *pyrE* mutant which needs to be corrected back to wild type once a mutant at a distal locus has been created. However, using ACE, this is rapid (5 days), and provides the opportunity to complement the mutant in parallel, through concomitant insertion of a wild type copy of the gene either under the control of its native promoter, or the strong *fdx* promoter.

Complementation is essential to unequivocally confirm the role of a genetic determinant in a specific phenotype [30]. Complementation is traditionally achieved using multicopy plasmids. The consequent abnormal gene dosage can, however, frequently fail to restore phenotype to wildtype levels. Thus, CwpV was over produced in a plasmid complemented *cwpV* mutant of *C. difficile* [31], the perfringolysin O titre of a *virR* mutant of the *C. perfringens* strain 56 was approximately 3-fold higher than the wild type strain when complemented with a multicopy plasmid carrying the *virRS* genes [32], while in a study of *Clostridium septicum* ‘haemolytic activity of the complemented strain was higher than the parent strain …‥ presumably a reflection of the presence of the *csa* gene on a multicopy plasmid.’ [33]. In the study of Dingle and co-workers [34], ‘although the wild-type 630Δ*erm* strain and the *fliC* mutant strain produced FliD, the *fliC*-complemented strain did not, suggesting that there was a reduction in *fliD* gene expression when FliC is expressed in trans off a plasmid rather than in *cis* from the chromosomal gene’. Similarly, plasmid complemented *agr* mutants of *Clostridium perfringens* failed to completely regain wild-type sporulation levels [35], a phenomenon attributed by the authors to ‘complementation involving a multicopy plasmid carrying the cloned *agr* locus’.

Ancillary effects can also arise when using plasmids for complementation studies due to the need to maintain plasmids, and the complementing gene, through supplementation with antibiotics. Thus, for instance, complementation of the a *sleC* mutation using a plasmid-located gene did not restore the phenotype to wild type [20], but did restore germination to the reduced level obtained in a vector only control. The presence of the plasmid alone, and most likely the effect of the added thiamphenicol needed to maintain the plasmid, detrimentally effected spore germination. Consequently, the use of plasmid complementation systems may require the inclusion of a vector only control [20]. This is not required when using ACE-based complementation at the *pyrE* locus. Crucially, the intrinsic stability of chromosomal complementation makes it the method of choice in those situations where plasmid maintenance through antibiotic supplementation is not a viable option, such as when complementation in an *in vivo* model is required.

The use of plasmids pMTL-YN1C and pMTL-YN2C positions the complementing gene in the genome immediately downstream of the *pyrE* gene (CD0187). This allows read through from the upstream promoter responsible for transcription of *pyrE*, the identity of which is unknown but may reside immediately upstream of CD0187 or, more likely, CD0184. The availability of transcriptional read through is important in those cases where the identity of the natural promoter of the complementation gene is not defined, and cannot be guaranteed to be cloned along with the structural gene, allowing, for instance, a promoter-less copy of the structural gene to be cloned. In those instances where the promoter is defined, and the operator wishes to exclude read through, a transcriptional terminator may simply be added during the cloning of the complementation gene and its promoter.

In certain instances it may be desirable to bring about ‘overexpression’ of the complementing gene. For this purpose, two further vectors were constructed (pMTL-YN1X and pMTLYN2X, for strains 630 and R20291, respectively) carrying the promoter of the ferredoxin gene (*fdx*) of *Clostridium sporogenes*. As a consequence of its central role in anaerobic electron transfer, the ferredoxin gene is highly expressed in *Clostridium*, with the ferredoxin protein representing, for instance, up to 2% of the soluble protein in *Clostridium pasteurianum* [36]. Indeed the *fdx* promoter of *C. perfringens* has been used to derive a highly effective clostridial expression system [37], while the *C. sporogenes fdx* promoter used here was employed to maximise expression of a nitroreductase gene in *C. sporogenes* [38].

Whichever vectors are used, their deployment involves only marginal additional effort compared to the use of autonomous complementation vectors. Thus, both methods (plasmid-based and ACE-based complementation) involve the assembly of the requisite vector in *E. coli* and its transformation into the appropriate clostridial mutant and the selection of transconjugants on antibiotic supplemented plates. ACE-mediated complementation merely requires re-streaking of the primary transconjugant onto minimal media lacking uracil. All those colonies that develop represent complemented strains in which the *pyrE* allele has been corrected to wildtype. The additional time required, therefore, equates to merely the 2 days needed for uracil prototrophic colonies to develop on the minimal media.

### Conclusion

We have demonstrated the use of a *pyrE*-based allele exchange system to make precise alterations to the genome of *C. difficile*. In using a *pyrE*- strain as the host, by whatever means a mutant is made (e.g., allelic exchange or ClosTron), the facility to insert a wild type copy of the gene at the *pyrE* locus provides a rapid mechanism for generating a stable complementation clone that is not compromised by inappropriate gene dosage effects. As such, the *pyrE* mutants made here are available to the scientific community for mutagenesis studies, along with the requisite *pyrE* correction vectors. The genomes of both strains have been subjected to genome re-sequencing and found to be devoid of any additional SNPs or Indels compared to the progenitor 630Δ*erm* strain or R20291 parental strains.

## Materials and Methods

### Bacterial strains and growth conditions

*Escherichia coli* TOP10 (Invitrogen) and *E. coli* CA434 [39] were cultured in Luria-Bertani (LB) medium, supplemented with chloramphenicol (25 µg/ml), where appropriate. Routine cultures of *C. difficile* 630 Δ*erm* [40] and *C. difficile* R20291 were carried out in BHIS medium (brain heart infusion medium supplemented with 5 mg/ml yeast extract and 0.1% [wt/vol] L-cysteine) [41]. *C. difficile* medium was supplemented with D-cycloserine (250 µg/ml), cefoxitin (8 µg/ml), lincomycin (20 µg/ml), and/or thiamphenicol (15 µg/ml) where appropriate. A defined minimal media [18] was used as uracil-free medium when performing genetic selections. A basic nutritive mannitol broth for growth assays of *C. difficile* strains were prepared as follows : Proteose peptone no. 2 4% [wt/vol] (BD Diagnostics, USA), sodium phosphate dibasic 0.5%[wt/vol], potassium phosphate monobasic 0.1%[wt/vol], sodium chloride, 0.2% [wt/vol], magnesium sulfate, 0.01% [wt/vol], mannitol, 0.6% [wt/vol] with final pH at +/−7.35. For solid medium, agar was added to a final concentration of 1.0% (wt/vol). *Clostridium sporogenes* ATCC 15579 was cultivated in TYG media [7]. All *Clostridium* cultures were incubated in an anaerobic workstation at 37°C (Don Whitley, Yorkshire, United Kingdom). Uracil was added at 5 µg/ml, and 5-Fluoroorotic acid (5-FOA) at 2 mg/ml. All reagents, unless noted, were purchased from Sigma-Aldrich.

### Conjugation into *C. difficile*

Plasmids were transformed into *E. coli* donor, CA434 and then conjugated into *C. difficile* [42]. Thiamphenicol (15 µg/ml) was used to select for *catP*-based plasmid. D-cycloserine (250 µg/ml) and cefoxitin (8 µg/ml) were used to counter select against *E. coli* CA434 after conjugation.

### DNA manipulations

DNA manipulations were carried out according to standard techniques. *C. difficile* genomic DNA for use in cloning and PCR analysis was prepared as described previously [9]. Polymerase chain reactions (PCR) used Failsafe™ PCR system (Epicentre) and Taq polymerase (Promega) in accordance with the manufacturers' protocols using primers detailed in Table 1. Primer design for amplification of DNA from *C. difficile* 630 Δ*erm* and R20291 strains were based on the available *C. difficile* genomes from the EMBL/GenBank databases with accession numbers AM180356 and FN545816 respectively. The oligonucleotides and the plasmids/strains used in this study are listed in Table 1 and Table 2, respectively. All DNA manipulations and cloning procedures were performed as per Sambrook [43].

**Table 1:** List of oligonucleotides used in this study.

Oligonucleotide	Binding site	Sequence (5′ to 3′)
Csp-pyrE-*Hpa*I-sF1	*pyrE* of C.*sporogenes*	AATATTGTTAACTAAGGAGAAGATATAAATGAGTAATATAAATGTTATAGATATATTAAAAGAATCAAAT
Csp-pyrE-*Hpa*I-sR1	*pyrE* of C.*sporogenes*	AATATGTTAACTTATTTTTGTTCTCTACTACCTGGTTTTACAAAAGGT
Cdi630-pyrD-sF1	*pyrD* of C. *difficile*	AGAGAAGGAATAAAAAGTTTAGACGAAATAAGAGG
λ6.5-sF2	6.5 kbp HindIII fragment of phage lambda, insert-specific reverse primer	TATGAGTACCCTGTTTTTTCTCATGTTCAGG
Cdi630-CD0189-sR3	CD0189 of C. *difficile*,chromosome-specific reverse primer	CCAAGCTCTATGACAGACAGCTCATTGTTTAGAAC
pyrE-int-SbfI-F	*pyrE* of *C. difficile* 630 Δ*erm* and R20291	CTGCAGGGGAGGGACATTTTTTATTATCTTCAGG
pyrEcomplement-Asc1-R	CD0188 of *C. difficile* 630 Δ*erm* and R20291	GGCGCGCCATAGTATATAACATTAATAAAATTTAAAATC
spo0A-YN-F2	upstream of *spo0A* of C. *difficile*	GGCAAGTATAAACTTGGATTATGGGTAAGAGAT
spo0A-YN-R2	downstream of *spo0A* of C. *difficile*	CTATATATCTTTCCATCTACAACTTCTATAG
cwp84-F3	upstream of *cwp84* of C. *difficile*	TTCTATAATTAATATGTACTCATAATCC
Cwp84-F4	knockout region of *cwp84*	CTGGACAAGCTACTTCAGGAG
Cwp84-R4	downstream of *cwp84* of C. *difficile*	CTGGACAAGCTACTTCAGGAG
mtlD-F3	upstream of *mtlD* of C. *difficile*	CTAGAGAATAGAATCGTGCTAGATTCAAATGAAG
mtlD-R3	downstream of *mtlD* of C. *difficile*	CTTTAACTGAATACTCTCTTGCCTTAG
mtlD-NotI-F	upstream of *mtlD*, inclusive of RBS and promoter sequence	GCGGCCGCTTTTTAATCACTCCTTATATTTTTATAC
mtlD-BamHI-R	end of coding sequence of *mtlD*	GGATCCTTATAAATTTTTCATAAATATATAACTTTTTTCGATATTATTTAAAAGTTCTTCG
mtlD-NdeI-F	start of *mtlD* coding sequence	CATATGAAAAAGGCAATTCAGTTTGGAGCAGG
Cpa-TT-Oligo-1	transcriptional terminator of the *Clostridium pasteurianum* ferredoxin gene	CTAGTATAAAAATAAGAAGCCTGCATTTGCAGGCTTCTTATTTTTATG
Cpa-TT-Oligo-2	transcriptional terminator of the *Clostridium pasteurianum* ferredoxin gene	CTAGCATAAAAATAAGAAGCCTGCAAATGCAGGCTTCTTATTTTTATA
spo0A-SpeI-F	*spo0A* gene of *C. difficile*	ATATACTAGTGGTATTTTTATAGATGAAATGATAAAATTGTAG
spo0A-BamHI-R	*spo0A* gene of *C. difficile*	ATATGGATCCTCAGTTTACAACTTGTAAAGACAC

**Table 2:** List of strains and plasmids used in this study.

Strains/Plasmids	Relevant features	Source
E. *coli* Top10	*mcrA* Δ(*mrr-hsdRMS-mcrBC*) *recA1*	Invitrogen
E. *coli* CA434	*hsd20*(r^B^-, m^B^-, *recA13, rpsL20, leu, proA2*, with IncPβ conjugative plasmid R702	[31]
C. *difficile* 630Δ*erm*	Erythromycin sensitive strain of strain 630	[34]
CRG1496	C. *difficile* 630Δ*erm* Δ*pyrE*	This study
CRG2547	Derived from CRG1496, in-frame deletion within *spo0A*, *pyrE* repaired to wildtype sequence	This study
CRG2302	Derived from CRG1496, in-frame deletion within *cwp84*, *pyrE* repaired to wildtype sequence	This study
CRG2445	Derived from CRG2302, *in situ* complementation of Δ*cwp84* with a single nucleotide change, *pyrE* repaired to wildtype sequence	This study
CRG2923	Derived from CRG1496, in-frame deletion within *mtlD*, *pyrE* repaired to wildtype sequence	This study
CRG2536	*C. difficile* 630_*erm mtlD*::intron *ermB*	This study
CRG2926	Derived from CRG2923, chromosomal complementation of *mtlD* with its natural RBS and promoter region, downstream of *pyrE*	This study
CRG2929	Derived from CRG2923, chromosomal complementation of *mtlD* preceded by the promoter region (P*_fdx_*) of the *Clostridium sporogenes* ferredoxin gene, downstream of *pyrE*	This study
C. *difficile* R20291 wild-type	BI/NAP1/027 Stoke Mandeville (2004–2005) isolate	Anaerobe Reference Laboratory, Cardiff, Wales, United Kingdom
CRG2359	C. *difficile* R20291 Δ*pyrE*	This study
CRG2548	Derived from CRG2359, in-frame deletion within *spo0A*, *pyrE* repaired to wildtype sequence	This study
CRG2549	Derived from CRG2359, in-frame deletion within *cwp84*, *pyrE* repaired to wildtype sequence	This study
CRG3059	Derived from CRG2549, *in situ* complementation of Δ*cwp84* with a single nucleotide change, *pyrE* repaired to wildtype sequence	This study
		
pMTL-YN18	same as pMTLJH18::λ6.5, but without the λ6.5 fragment, homology arms are specific to R20291 sequence and pCB102 replicon replaced with pBP1	This study
pMTL-ME2	Derived from pMTL83151 through addition of a C. *sporogenes pyrE* and *C. perfringens catP* gene. Carries an unaltered pCB102 replicon, which includes a NsiI site in the *repH* gene.	This study
pMTL-YN3	Derived from pMTL-ME2 by blunt-end ligation of the NsiI site within *repH*, causing a frame-shift in RepH	This study
pMTL-YN3::630spo0A*	Same as pMTL-YN3, *spo0A* KO cassette cloned into SbfI/AscI sites	This study
pMTL-YN3::630cwp84*	Same as pMTL-YN3, *cwp84* KO cassette cloned into SbfI/AscI sites	This study
pMTL-YN3::630cwp84c	Same as pMTL-YN3, full sequence of cwp84 with single base change cloned into SbfI/AscI sites	This study
pMTL-YN4	Derived from pMTL-YN3 by replacing the pCB102 replicon with that of pBP1	This study
pMTL-YN4::R20291spo0A*	Same as pMTL-YN4, *spo0A* KO cassette cloned into SbfI/AscI sites	This study
pMTL-YN4::R20291cwp84*	Same as pMTL-YN4, *cwp84* KO cassette cloned into SbfI/AscI sites	This study
pMTL-YN4::R20291cwp84c	Same as pMTL-YN4, full sequence of *cwp84* with single base change cloned into SbfI/AscI sites	This study
pMTL-YN3::630mtlD*	Same as pMTL-YN3, *mtlD* KO cassette cloned into SbfI/AscI sites	This study
pMTL-YN1	same backbone as JH-18 but without the 6.5kb fragment of λphage DNA, entire *pyrE* coding sequence minus the first 50 bases	This study
pMTL-YN1C	same as pMTL-YN1, except for a MCS inserted after *pyrE* gene to enable cloning of target gene for purpose of complementation	This study
pMTL-YN1C::mtlD	same as pMTL-YN1C, with the full coding sequence of mtlD and its natural RBS cloned into the *Nde*I and *BamH*I sites	This study
pMTL-YN1X	same as pMTL-YN1C, for a *fdx* promoter inserted after *pyrE* gene and before the MCS to enable overexpression of target gene	This study
pMTL-YN1X::mtlD	same as pMTL-YN1C::mtlD, with the full coding sequence of *mtlD* cloned downstream of the fdx promoter into the *Nde*I and *BamH*I sites	This study
pMTL-YN2	same as pMTL-YN1, but with homology arms specific to R20291 sequence and pCB102 replicon replaced with pBP1	This study
pMTL-YN2C	same as pMTL-YN1C, but with homology arms specific to R20291 sequence and pCB102 replicon replaced with pBP1	This study
pMTL-YN2X	same as pMTL-YN1X, but with homology arms specific to R20291 sequence and pCB102 replicon replaced with pBP2	This study

### Vector Construction

Plasmid pMTL-YN18 (accession number; JX465728), used to create a *pyrE* ^−^ mutant in R20291, is an equivalent vector to pMTL-JH18 [9] in which the pCB102 replicon was replaced with that of pBP1 (isolated from pMTL82251), using the flanking AscI and FseI sites specifically created for this purpose [17]. Plasmid pMTL-YN1 (accession number; JX465729) was made by cloning a 1,753 bp SbfI/AscI fragment between the corresponding restriction sites of plasmid pMTL-JH20 [9]. This fragment was generated from the 630Δ*erm* genome by PCR using the primers pyrE-int-SbfI-F and pyrEcomplement-Asc1-R, and carries a truncated copy of *pyrE*, lacking the first 50 nucleotides of the structural gene, together with 1200 bp from immediately 3′ to the stop codon of *pyrE*, which includes the entire coding sequence of CD0188 of 630Δ*erm*. To generate the equivalent *pyrE* correction vector (pMTL-YN2, accession number; JX465732) for R20291, the pCB102 replicon of pMTL-YN1 was replaced with that of pBP1 from pMTL82251 (using the AscI and FseI sites) and the 630Δ*erm* DNA between the SbfI and AscI sites replaced with the equivalent region from the R20291 genome. In this case, the DNA was synthesized by DNA2.0 specific to the R20291 chromosome by PCR as two contiguous SbfI/NdeI and NdeI/AscI fragments. The PyrE complementation vector pMTL-ME2 was constructed by inserting the *C. sporogenes* ATCC 15579 *pyrE* gene into pMTL83151. The *pyrE* gene was amplified by PCR from genomic DNA prepared from *Clostridium* sporogenes ATCC 15579 using primers Csp-pyrE-*Hpa*I-sF1 and Csp-pyrE-*Hpa*I-sR1. The PCR product was digested with HpaI and gel purified followed by cloning in same orientation as *catP* in pMTL83151 [17] linearized with the same enzyme.

To construct the ACE overexpression plasmids pMTL-YN1X (accession number; JX465731) and pMTL-YN2X (accession number; JX465734), a 214 bp NotI/NdeI fragment encompassing the *C. sporogenes fdx* promoter was isolated from plasmid pMTL82253 and inserted between the equivalent sites of pMTL-JH14 [9]. The plasmid obtained (pMTL-JH14::Pcspfdx) was cleaved with NheI, and ligated to two, annealed oligonucleotides (Cpa-TT-Oligo-1 and Cpa-TT-Oligo-2) which encompassed the transcriptional terminator of the *Clostridium pasteurianum* ferredoxin gene. A 517 bp NotI/NheI fragment (encompassing the *fdx* promoter, *lacZ'* and multiple cloning sites, and the transcriptional terminator of the *C. pasteurianum* ferredoxin gene) was then isolated from the resultant plasmid (pMTL-ME6X) and inserted between the equivalent sites in pMTL-YN1 and pMTLYN2, to yield pMTL-YN1X and pMTL-YN2X, respectively. In parallel, pMTL-ME6X was digested with EcoRI and NheI and the released 293 bp fragment inserted between the equivalent sites of pMTL-JH14 [9] to make pMTL-ME6C. This plasmid was then cleaved with NotI and NheI, and the released 323 bp fragment inserted between the equivalent sites of pMTL-YN1 and pMTLYN2, to yield the complementation vectors pMTL-YN1C (accession number; JX465730) and pMTL-YN2C (accession number; JX465733), respectively.

### Allelic Exchange Cassettes

Allele exchange cassettes were assembled, specific to the three target loci (*spo0A*, *cwp84* and *mtlD*) of the two strains, composed of a left-hand homology arm (LHA) and a right-hand homology arm (RHA), each of approximately 500 to 800 bp in size. Each cassette was cloned between the *Sbf*I and *Asc*I restriction recognition sites in pMTL-YN3 (accession number; JX465735) to create pMTL-YN3::630spo0A*, pMTL-YN3::630cwp84* and pMTL-YN3::630mtlD*. Similarly, pMTL-YN3::R20291spo0A* and pMTL-YN3::R20291cwp84* were made by cloning the allelic exchange cassettes between the *Asc*I and *Sbf*I sites in pMTL-YN4 (accession number; JX465736).

The *spo0A* knockout cassette was synthesized by DNA2.0 which consists of a 672 bp LHA (bases 1412012 to 1412789 on the forward strand of *C. difficile* 630 Δ*erm* genome) fused to a 800 bp RHA (bases 1413275 to 1414074 on the forward strand of *C. difficile* 630 Δ*erm* genome) which were designed to make an in-frame deletion removing codons 65 to 226, inclusive, out of a total of 275 codons). For the equivalent in *C. difficile* R20291, a 778 bp LHA (bases 1319650 to 1320427 on the forward strand of the *C. difficile* R20291 genome) was fused to a 800 bp RHA (bases 1320913 to 1321712 on the forward strand of the *C. difficile* R20291 genome). Each cassette was designed to incorporate a *Sbf*I site at the 5′ end and an *Asc*I site at the 3′ end to facilitate cloning into the pMTL-YN3 plasmid. The *cwp84* knockout cassette consists of a 653 bp LHA (bases 1045907 to 1046559 on the reverse strand of the *C. difficile* 630 Δ*erm* genome) fused to a 558 bp RHA (bases 1047778 to 1048335 on the reverse strand of the *C. difficile* 630 Δ*erm* genome) which were designed to remove codons 214 to 629, inclusive, representing the removal of 417 codons from a total of 814). For the equivalent in *C. difficile* R20291, a 713 bp LHA (bases 1067119 to 1067771 on the reverse strand of the *C. difficile* R20291 genome) was fused to a 558 bp RHA (bases 1068990 to 1069547 on the reverse strand of the *C. difficile* R20291 genome). The *mtlD* knockout cassette consists of a 654 bp LHA (bases 1593564 to 1594217 on the reverse strand of the *C. difficile* 630 Δ*erm* genome) fused to a 654 bp RHA (bases 1595353 to 1596006 on the reverse strand of the *C. difficile* 630 Δ*erm* genome) which were designed to make an in-frame deletion between codon 3 and codon 387 of *mtlD* (total codons 388).

### Complementation Vectors

Plasmids for the complementation of the *spo0A* mutants of strains R20291 and 630Δ*erm* were constructed as follows. The *spo0A* of strain *C. difficile* 630Δ*erm* was PCR amplified as a 1216 bp DNA fragment using the two primers spo0A-SpeI-F and spo0A-BamHI-R and cloned in pCR2.1 vector (Invitrogen) and verified by sequencing. The *spo0A* was re-isolated as a 1.2 kb EcoRI and BamHI fragment and inserted between the equivalent sites of pMTL-YN2C to give the R20291 complementation plasmid pMTL-YN2C*spo0A*. To derive an equivalent plasmid for strain 630Δ*erm*, the *spo0A* gene was excised from pMTL-YN2C*spo0A* as a 1.2 kb DNA fragment following cleaved with NotI and BamHI and inserted into the appropriate sites of pMTL-YN1C to give the complementation plasmid pMTL-YN1C*spo0A*.

To generate the two required allelic exchange cassettes need for complementation of the *cwp84* in-frame deletions, the portion of *cwp84* missing in the two plasmids (pMTL-YN4::R20291cwp84* and pMTL-YN3::630cwp84*) originally used to make the in-frame deletions were synthesised (DNA2.0) as a 1,082 bp NdeI-KpnI fragment (R20291) and a 1,255 bp NdeI-XbaI fragment (630Δ*erm*) generated by cloned into the appropriate allelic exchange vectors to yield plasmids pMTL-YN4::R20291*cwp84^c^* and pMTL-YN3::630*cwp84^c^*. In order to be able to distinguish the complementation allele from wild type, the synthesised region included a single nucleotide change (T to A) to base 2280 of *cwp84* which retains the codon specificity (codon 760, Valine) but creates a ScaI restriction enzyme recognition site.

The complementation plasmid pMTL-YN1C*mtlD* was constructed by the PCR amplification of the *C. difficile* 630Δ*erm mtlD* gene, together with its 5′ non-coding region, as a 1,779 bp fragment (using primers mtlD-NotI-F and mtlD-BamHI-R), and its subsequent insertion, following cleaved with NotI and BamHI, between the NotI and BamHI sites of pMTL-YN1C. pMTL-YN1X*mtlD* was derived by the PCR amplification of the *C. difficile* 630Δ*erm mtlD* structural gene alone (ie., lacking its native promoter), as a 1,161 bp fragment (using primers mtlD-NdeI-F and mtlD-BamHI-R), cleaved with NdeI and BamHI, and insertion between the NdeI and BamHI sites of pMTL-YN1X. This positioned the *mtlD* gene under the transcriptional and translational (RBS) control of *fdx* promoter.

### ACE Procedure

The procedure adopted was as previously described [9]. For inactivation of *pyrE*, *E. coli* CA434 donor cells carrying pMTL-YN18 were conjugated with R20291 and transconjugants selected on BHIS media supplemented D-cycloserine (250 µg/ml), cefoxitin (8 µg/ml), thiamphenicol (15 µg/ml) and uracil (5 µg/ml). A single transconjugant was re-streaked onto the same medium and then a ‘large’ representative colony streaked onto minimal agar medium agar supplemented with FOA (2 mg/ml) and uracil (5 µg/ml). The colonies that arose were re-streaked twice onto the same media, and analyzed by PCR to confirm deletion of *pyrE* (as detailed in Results), and Sanger sequencing was used to confirm the expected genotype. Confirmation that the plasmid had been lost, was obtained by patch plating onto BHIS agar supplemented with thiamphenicol and establishing that no growth occurred. For correction of the *pyrE* mutation (ie., plasmids pMTL-YN1, 1C, 1X, 2, 2C and 2X) transconjugants were streaked onto minimal media without uracil or FOA supplementation and those colonies that developed analysed as above.

### Allelic exchange procedure

The adopted protocol resembles that used for the isolation of allelic exchange mutants using *codA* as a negative selection marker [8], except counter-selection was based on differential resistance to FOA rather than FC. It in essence involves the initial isolation of pure single crossovers clones, and their subsequent transfer onto selective plates to identify those cells in which the plasmid has excised. These clones correspond to colonies that are resistant to FOA as a consequence of loss of the heterologous PyrE+ allele and reversion to a *pyrE* minus phenotype. Depending which homology arm undergoes recombination, plasmid excision can result in either the desired double crossover mutant, or a wild type cell. In the absence of any bias, the wild type cells and desired mutants should be present in equal proportions, ie., 50∶50.

Each plasmid was conjugated into the appropriate host and plated onto BHIS media (supplemented with cycloserine, cefoxotin, thiamphenicol and uracil) and the transconjugants restreaked onto fresh media to allow the identification of putative single crossover mutants. These were readily identified as visibly larger colonies after 16–24 hr. Such colonies were sequentially restreaked from single colonies and after 2–4 passages, their identify and purity confirmed by an appropriate PCR. Unlike ACE, where powerful selection allows the purification of the desired double crossover excision event, it is crucial that the single cross-over integrants are pure, and not contaminated with wild type cells, ie., cells in which integration has not occurred. Otherwise, the contaminating wild type cells, which are all resistant to FOA, will cause unacceptable high background when cells are plated on FOA media during the screen for the desired double crossover events. To establish that no wild type cells were present, an appropriate primer pair (spo0A-YN-F2/spo0A-YN-R2 in the case of *spo0A*, cwp84-F3/cwp84-R4 in the case of *cwp84*, and mtlD-F3/mtlD-R3 the case of *mtlD*) was used that annealed to the chromosome at positions that flanked the site of the in-frame deletion but that were both distal to the regions that comprised the homology arms of the plasmid borne allelic exchange cassette. The absence of a DNA product of a size consistent with the wild type indicated clonal purity, and that wild type cells were not present. To confirm that they were indeed single crossover integrants, two different primers (M13F and M13R) were used that annealed to plasmid specific sequences together with the appropriate flanking primers. The presence of a DNA fragment indicated that the clones were indeed single crossover integrants, while the size was diagnostic of at which homology arm (LHA or RHA) recombination had occurred.

Following the isolation of pure single crossover integrants of the two strains, a single colony was re-streaked onto minimal medium supplemented with FOA and uracil to select for cells in which the integrated plasmid had excised. The isolated FOA resistant colonies were then screened by PCR using primers that anneal to the upstream and the downstream sequence of the respective target. DNA products of the predicted size were subjected to Sanger sequencing to confirm their genotype.

### Cwp84 Analysis Methods

S-layer extracts from the WT, mutant and complemented strains were prepared by the glycin acid method [44]. Proteins were separated by SDS-PAGE and transferred onto a PVDF membrane for immunoblotting. Blocking of the membrane was followed by incubation with either anti-Cwp84 (1/2,000 dilution) or anti-HMW-SLP (1/10,000 dilution). Washing was done as previously described [45], and bound antibodies were detected using goat anti-rabbit immunoglobulin G alkaline phosphatase conjugate (1/20,000 dilution; Sigma) with the substrates nitroblue tetrazolium and 5-bromo-4-chloro-3-indolylphosphate (Sigma). Anti-HMW-SLP antibodies were a kind gift from Pr. Neil Fairweather, Imperial College, London.

## Supporting Information

File S1 **Supporting Information.**
(DOCX)
