# Supplementary material for: Expanding the Repertoire of Gene Tools for Precise Manipulation of the Clostridium difficile Genome: Allelic Exchange Using pyrE Alleles
Source: PLoS One. 2013 Feb 6;8(2):e56051. doi: 10.1371/journal.pone.0056051 (PMC3566075; doi:10.1371/journal.pone.0056051)
Supplement: File S1 — Supporting Information. (DOCX) [file pone.0056051.s001.docx]

**Expanding the Repertoire of Gene Tools for Precise Manipulation of the *Clostridium difficile* Genome: Allelic Exchange Using PyrE Alleles.**

**SUPPORTING INFORMATION**

Yen Kuan Ng^≠ 1^, Muhammad Ehsaan^≠ 1^, Sheryl Philip^1^, Mark Collery^1^, Clare Janoir^2^, Anne Collignon^2^, Stephen T. Cartman^1^, Nigel P. Minton^1^*

^≠^ These authors contributed equally to this work.

**Author affiliations:**

^1^Clostridia Research Group, NIHR Biomedical Research Unit in GI Disease, Centre for Biomolecular Sciences, University Park, University of Nottingham, Nottingham, NG7 2RD, United Kingdom. ^2^Université Paris-Sud, Faculté de Pharmacie, Département de Microbiologie, Unité EA 40-43, 92296 Châtenay-Malabry Cedex, France.

*To whom correspondence should be addressed: Nigel P. Minton

Clostridia Research Group, Centre for Biomolecular Sciences, University Park, University of Nottingham,

Nottingham, NG7 2RD, United Kingdom

Phone: +44 (0)115 84 67458

Fax: +44 (0)115 82 32120

**(a)**

**(b)**


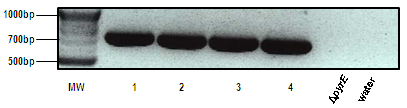


**Figure S1. PCR screening of ACE generated *pyrE* mutant and wildtype strains in *C. difficile* strains 630∆*erm* and *C. difficile* R20291.** (a) five random FOA^R^, *pyrE* mutants (lanes 1-5) made in R20291 using pMTL-YN18, and; (b) five random *pyrE* repaired clones (lanes 1-5) from R20291 made using pMTL-YN2. MW is a 2-Log DNA Ladder (NEB) molecular weight marker, plasmid is a pMTL-YN18 DNA control, WT is a wild-type *C. difficile* DNA control, and ∆*pyrE* is a mutants control taken from the strain in lane 1 of (a). The primers used, and expected size of the DNA fragments generated were: (a) primers pyrE-F2/pyrE-R2 and a 565 bp DNA fragment, and; (b) primers cdi630-pyrD-SF1/pyrE-R2 and a 750 bp DNA fragment.

FIGURE 10a

**Figure S2. PCR screening of double crossover candidate clones of *C. difficile* 630Δ*erm* for *spo0A*, *cwp84* and *mtlD*.** (A) PCR screening of four (1-4) putative *spo0A* in-frame deletion clones using primers spo0A-YN-F2 and spo0A-YN-R2. The desired mutant should give a DNA product of 1,845 bp in size (clone 2), while the wild type allele (clones 1, 3 and 4) generates a product of 2,331 bp in size. (B) PCR screening of three (1-3) putative *cwp84* in-frame deletion clones using primers cwp84-F3 and cwp84-R4. A double crossover mutant (clone 3) should give a PCR product of 1,418 bp in size, as opposed to the 2,636 bp fragment predicted for the wild type allele (clones 1 and 2). (C) PCR screening of three (1-3) putative in-frame deletions of *mtlD* using primers mtlD-F3 and mtlD-R3. The in-frame deletion mutant is predicted to yield a PCR DNA product 1,418 bp in size (clones 1 and 3), whereas the wild-type allele (clone 2) gives a DNA product of 2,582 bp in size. PCR product from wild-type or double-crossover wild-type revertants. MW is a 2-Log DNA Ladder (NEB) molecular weight marker and WT is a wild-type *C. difficile* DNA control.


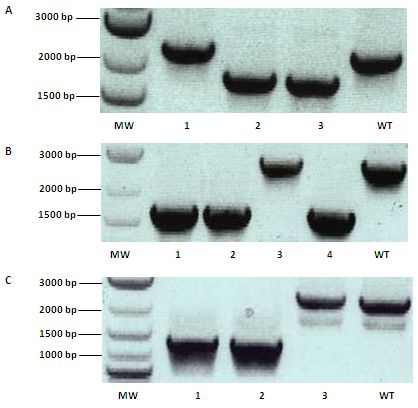


**Figure S3. PCR screening of double crossover candidate clones of *C. difficile* R20291 for *spo0A* and *cwp84.*** (A) PCR screening of three (1-3) putative *spo0A* in-frame deletion clones using primers spo0A-YN-F2 and spo0A-YN-R2. The desired mutant should give a DNA product of 1,845 bp in size (clones 2 and 3), while the wild type allele (clone1) generates a product of 2,331 bp in size. (B) PCR screening of four (1-4) putative *cwp84* in-frame deletion clones using primers cwp84-F3 and cwp84-R4. Double crossover mutants (clones 1, 2 and 4) should give a PCR product of 1,418 bp in size, compared to the 2,636 bp fragment predicted for the wild type allele (clone 3). MW is a 2-Log DNA Ladder (NEB) molecular weight marker and WT is a wild-type *C. difficile* DNA control.


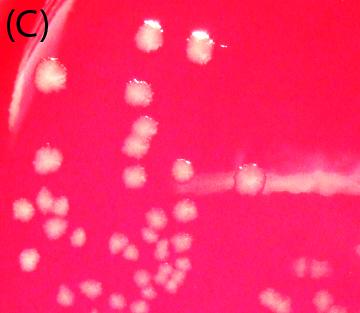

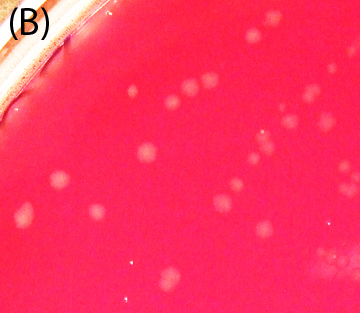

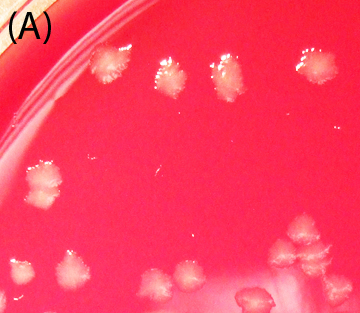


**Figure S4. Comparison of colony morphology of *C. difficile* 630Δerm strains.** *C. difficile* 630Δerm (A) 630ΔermΔ*cwp84* mutant (B), and 630ΔermΔ*cwp84*-complemented (C) were streaked onto anaerobic blood agar plate and incubated overnight to observe colony morphology. Colonies of *C. difficile* 630Δerm (A) and 630ΔermΔ *cwp84*-complemented (C) are indistinguishable and showed irregular edged colonies compared to *C. difficile* 630Δerm cwp84 mutant (B) showed more rounded colonies and are noticeably smaller due to its slower growth rate.





**Figure S5. Complementation of *spo0A* mutants of *630Δerm*∆*pyrE* and R20291∆*pyrE*.**  The sporulation phenotype of the mutants and complemented mutants were compared to the wildtype strains by assaying colony forming units (CFU) on BHIS supplemented with 0.1% [w/v] sodium taurocholate before and after heat shock (65°C for 30 min) following growth of each strain in BHIS media for 120 h. Assayed strains are: 630∆*erm*∆*pyrE*∆*spo0A*; 630∆*erm**(*spo0A*); 630∆*erm*; R20291∆*pyrE*∆*spo0A­*; R20291*(*spo0A*), and; R20291, where * indicates the *spo0A*-complemented strain (*spo0A*) which has had a copy of the *spo0A* gene inserted into the chromosome, concomitant with the correction of the *pyrE* allele back to wildtype. The detection limit for the assay was 50 CFU/ml. All experiments were undertaken in triplicate. Phase contrast microscopy confirmed that phase-bright spores were absent in the mutant cultures but present in both the complemented and wildtype strains.

**Fig. S6. Growth of *C. difficile* 630Δerm strains with mannitol as the sole carbon source.**. (B) The growth of ∆*mtlD* was limited in mannitol broth, while growth of the ∆*mtlD* complemented and *mtlD* overexpressed strains were restored to wildtype levels. (C) The pH of the media broth showed a dip in pH caused by the fermentation of mannitol for the wildtype, ∆*mtlD* complemented and ∆*mtlD* overexpressed strains, which correlate to their growth. The 630 ∆*erm* ∆*mtlD* mutant strain grew very weakly in mannitol broth, which was reflected in the sustained pH levels of the media. Experiments were undertaken in triplicate.
